# The Effect of Physical Activity on Drug Cravings of Drug Addicts With AIDS: The Dual Mediating Effect of Internal Inhibition

**DOI:** 10.3389/fpsyg.2020.02002

**Published:** 2020-10-02

**Authors:** Tingran Zhang, Kun Wang, Meichen Qu, Haonan Jiang, Xi Chen, Jiong Luo

**Affiliations:** ^1^Research Centre For Exercise Detoxification, College of Physical Education, Southwest University, Chongqing, China; ^2^Chongqing Municipal Public Security Bureau, Chongqing, China

**Keywords:** drug addicts with AIDS, physical activity, internal inhibition, drug craving, drug-taking years, dual mediating effect

## Abstract

**Background:**

As a global infectious disease, Acquired Immune Deficiency Syndrome (AIDS) poses a serious threat to the safety and health of the society. In recent years, the proportion of drug addicts infected with HIV has been increased, and drug addicts became one of the main carriers of the spread of AIDS, which has attracted worldwide attention. It has been reported that physical activity has positive effects on improving the inhibitory function of drug addicts and reducing their drug craving, but the mechanism of the internal inhibition remains to be further explored.

**Method:**

The drug addicts in an AIDS treatment center in Chongqing were investigated by means of a Physical Activity Rating Scale (PARS – 3), Internal Inhibition Scale and Drug Craving Scale, and a structural equation model was established.

**Results:**

(1) There is no gender difference in the internal inhibition and drug craving of drug addicts with AIDS, and there are significant differences across the types and years of drug abuse. (2) The amount of physical activity in drug addicts with AIDS is positively correlated with the intrinsic inhibition, while the internal inhibition is negatively correlated with the drug craving, and the physical exercise amount is negatively correlated with the drug craving. The years of drug abuse were negatively correlated with internal inhibition and positively correlated with drug craving. (3) Internal inhibition plays a partly mediatory role between the physical activity amount and the craving for drugs, and internal inhibition plays a partly mediatory role between the number of years of drug abuse and the craving for drugs, which indicates that internal inhibition has a dual mediating effect.

**Conclusion:**

Actively participate in physical activity, especially high-intensity physical activity, can effectively enhance the internal inhibition of drug addicts with AIDS and reduce their drug craving. Meanwhile, the difference in the drug-taking years among addicts should be paid attention to, and the physical activity prescription should be formulated according to the actual situation.

## Introduction

Acquired Immune Deficiency Syndrome (AIDS) is a malignant infectious disease caused by the HIV virus and the subsequent damage to the immune system of the body, which can cause serious harm to humans (Zhang et al., 2018). It is mainly transmitted through unprotected sex, the sharing of unclean syringes, and high-risk behaviors such as drug abuse, which tend to occur in people with deep contact or specific relationships (Li et al., 2015). Among the mentioned methods, injecting drug use (IDU) is the most common way to spread HIV (Li et al., 2013). It is reported that drug users have a higher risk of HIV infection than the general population due to their high-risk behaviors such as unsafe sex and needle sharing (Xu et al., 2014). Globally, in 2016, about 275 million people have taken drugs at least once, accounting for 5.6% of the global population within the age of 15–64 (UNODC, 2018), and the proportion of drug abuse cases of AIDS continues to rise, which poses a considerable threat to social security (Chen G. H. et al., 2017). Studies have shown that the higher the degree of dependence on drugs people have, the more severely their inhibition ability will be impaired, and drug addicts are more likely to engage in unsafe sexual behaviors, needle sharing, and other dangerous behaviors. Thus they are easily becoming a high-risk population for the transmission of AIDS (De Los Cobos et al., 2011; Xu et al., 2014). Therefore, we speculate that the inhibition ability of drug addicts with AIDS may be related to their drug cravings and unsafe behaviors.

### Physical Activity and Drug Craving

Drug craving is an intense and uncontrollable craving for drug users to regain the psychoactive substances that they have experienced, causing them to subconsciously over-focus on drug-related cues and continue to use addictive drugs regardless of severe consequences (Yu et al., 2002; Field et al., 2009). Persistent and intense cravings can not only skew an addict’s attention but also subconsciously focuses too much on drug-related cues, which is the main reason for compulsive drug use (Field et al., 2009; Schnell et al., 2013; Serre et al., 2015). The higher the drug craving a person has, the higher the level of anxiety and depression (De Los Cobos et al., 2011), and the stronger the urge to use drugs and participate in corresponding drug-seeking behavior will be. Therefore, how to effectively reduce the craving for drugs is an urgent scientific problem in the treatment of drug addiction. Interestingly, in recent years, the scientific concept of *exercise is a good doctor* has gradually gained popularity. As an environment-friendly rehabilitation method, exercise has attracted much attention in the rehabilitation effect of drug addiction treatment. A study found that after active aerobic exercise intervention for people who smoked marijuana and opioid, drug withdrawal patients’ drug cravings were significantly reduced, and their withdrawal symptoms were significantly improved (Roessler, 2010). After aerobic exercise intervention, the cardiopulmonary endurance of the person undergoing drug rehabilitation was enhanced considerably and accompanied by a marked decrease in drug craving (Brown et al., 2010). A review of studies has shown that physical exercise can effectively inhibit the psychological craving and the corresponding relapse behavior of addicts through the regulation of neurotransmitters and hormones (Zhao et al., 2018). After 3 months of exercise interventions such as cycling, jogging, or skipping, the drug craving of methamphetamine addicts showed a significant downward trend (Wang and Zhu, 2017). Therefore, the hypothesis H1 is proposed in this study: physical activity is negatively correlated with drug cravings of drug addicts with AIDS.

### Internal Inhibition and Drug Craving

Long-term use of addictive medicines, such as drugs, will severely damage the cognitive function of drug users’ brains, resulting in abnormal activation of the prefrontal cortex, which will lead to abnormal cognition and behavior (Yin et al., 2012). As an important branch of brain cognitive function, internal inhibition plays an important role in inhibiting the urge and craving for drugs. Internal inhibition refers to the ability to cancel a dominant response or to stop an inappropriate or irrelevant action, which is an important part of the executive function of the brain, and is clinically closely related to the prevention and treatment of tobacco addiction, schizophrenia, and drug abuse (Friedman and Miyake, 2003; Taylor et al., 2007). Studies have shown that internal inhibition is closely related to drug craving (Yang et al., 2007). In the field of drug addiction research, drug addicts with low self-control are more likely to commit to drug cravings and corresponding abuse behaviors, while those with high self-control show fewer abuse behaviors (Tarantino et al., 2015). Among drug addicts with AIDS, studies found that the cravings of drug addicts was negatively correlated with the internal inhibition, that is, the higher the drug craving was, the lower the internal inhibition people had (Liu, 2008). Based on this issue, the hypothesis H2 was proposed in this study: the internal inhibition of drug addicts with AIDS was negatively correlated with drug craving.

### Physical Activity and Internal Inhibition

Research shows that physical activity is closely related to internal inhibition. Among non-drug-addicts, aerobic exercise can reduce the activation level of the anterior cingulate cortex in the elderly and the prefrontal lobe in children, as well as improve the central executive function (Colcombe et al., 2004; Chaddock-Heyman et al., 2013; Corona et al., 2016; Guicciardi et al., 2019), which contributes to the improvement of its internal inhibitory capacity. However, the role of physical exercise in enhancing the internal inhibitions for drug addicts has also been emphasized. Hillman and Drobes (2012) believes that active participation in aerobic exercise can repair the impaired cognitive control ability of drug addicts to a certain extent, enhance the ability to suppress the urge to use drugs, and thus to reduce the withdrawal symptoms and play a role in rehabilitation. Empirical studies have shown that the degree of physical activity participation has a significant impact on the general inhibition and drug-related internal inhibition of drug addicts (Zhao et al., 2017). In fact, both acute exercise and aerobic exercise can improve the inhibitory function of methamphetamine addicts. Meanwhile, exercise intensity and amount of exercise are of great significance to the inhibition and control of addicts and the improvement of emotional symptoms (Rawson et al., 2015; Wang D. S. et al., 2015). However, for drug addicts with AIDS, the effect of physical exercise on the internal inhibition remains to be further explored. Therefore, this study proposed hypothesis H3: physical activity is positively correlated with the internal inhibition of drug addicts with AIDS.

### Mediation Effect of Internal Inhibition

**FIGURE 1:**
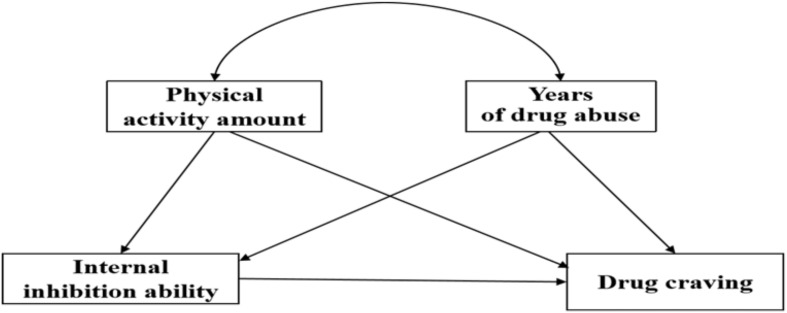
Intermediary hypothesis model diagram.

In conclusion, active physical activity can not only regulate the emotional state but also relieve the withdrawal reaction of drug addicts (Wang and Zhu, 2017; Gong et al., 2019). Moreover, it can also improve the functional brain areas such as inhibition and cognition areas, effectively inhibit the drug impulse and corresponding drug-seeking behavior of addicts, so that to achieve the purpose of reducing the drug craving (Hillman and Drobes, 2012; Wang et al., 2016), which fully reflects that the internal inhibition plays an important role in the relationship between physical exercise and drug craving. Similar studies have found that smoking dependence on college students’ self-control has a mediation role between physical activity and smoking dependence (Zhu et al., 2014). Among the drug-dependent populations, Wang D. S. et al. (2015) suggested that enhanced inhibitory force induced by acute exercise is a possible mechanism for the relationship between acute exercise and drug craving. Active aerobic exercise can significantly improve the performance of inhibitory brain regions and suppression capabilities by stimulating the brain nerves of methamphetamine addicts, and significantly improve the performance of addicts in the suppression task, thereby reducing the drug cravings of addicts (Wang et al., 2016; Chen et al., 2019). However, at present, there is a lack of substantive research on the relationship between physical activity, internal inhibition, and drug craving of drug addicts with AIDS in academia. Besides, studies have shown that drug addiction year is an important factor affecting drug addicts’ internal inhibition and drug craving (Jiang, 2006). Among non-AIDS-infected drug addicts, studies have shown that addicts will develop a stronger sense of drug dependence and gradually increase their physical tolerance to drugs after taking drugs for a long time (Chen G. H. et al., 2017). For drug addicts with AIDS, it is unknown what changes will occur in their internal inhibition and drug craving with the increase of drug-taking years. Preacher and Hayes (2008) believe that in most cases, independent variables can not only directly affect the dependent variable, but also indirectly affect the dependent variable through one or more mediation variables, and regression and structural equation models are usually used when measuring indirect effects. Baron and Kenny (1986) pointed out in an early study that testing the mediation effect mainly includes three steps: first, the independent variable has an effect on the dependent variable, and the regression coefficient reaches significance; Second, the independent variable has an effect on the mediation variable, and the regression coefficient reaches significance; Third, independent variables and intermediary variables can together significantly affect the dependent variable. Therefore, this study hypothesized that H4: the internal inhibition has a mediating effect between physical activity and drug craving in drug addicts with AIDS; Hypothesis H5: internal inhibition has a mediating effect between the drug-taking and drug craving in drug addicts with AIDS. The mediation hypothesis model diagram is shown in Figure 1.

## Participants and Methods

### Participants

This study adopts cluster sampling methods to conduct the survey to all detoxification addicts in the Education and Correction Center for Drug Abusers with AIDS in Chongqing, China. In order to ensure the validity and reliability of the questionnaires, the supervision personnel of the drug control center will distribute the questionnaires on their behalf, and the dormitory units will fill in and collect the questionnaires uniformly. Before filling out the questionnaires, all drug addicts will sign the informed consent form (Table 1). Among which, a total of 420 questionnaires were sent out, and 409 were collected, with a collection rate of 97.38%. Fourteen invalid questionnaires were excluded, and 395 remained, with an effective rate of 96.57%. Among the AIDS transmission routes, 302 (51.89%) used needles for drug sharing, 215 (36.94%) had sexual promiscuity, and 75 (12.89%) received other routes.

**TABLE 1:** Basic characteristics of respondents (*N* = 395).

**Demographic variable**	**Category**	***X* ± *SD* / n (%)**	**Drug-related data**	**Category**	***X* ± *SD* / n (%)**
Age (years)		35.79 ± 9.91	Drug group	New drug	145 (36.71%)
Body height (m)		1.59 ± 0.05		Traditional drug	144 (36.46%)
Bodyweight (kg)		57.78 ± 8.12		Mixed drug	106 (26.84%)
Gender	Men	160 (40.51%)	Mainly drug used	Cannabis	34 (8.61%)
	Women	235 (59.49%)		Heroin	88 (22.28%)
Educational level	Primary school	56 (14.18%)		Cocaine	41 (10.38%)
	Middle school	178 (45.06%)		Methamphetamine	112 (28.35%)
	High school	130 (32.91%)		K powder	68 (17.22%)
	College or above	31 (7.85%)		Others	52 (13.16%)
Occupation	Unemployed	136 (34.43%)	Relapse	Once	72 (18.23%)
	Self-employed	108 (27.34%)		Twice	143 (36.20%)
	Office clerks	101 (25.57%)		Three times or more	180 (45.57%)
	Civil servant	50 (12.66%)	Drug use years		7.93 ± 7.40
				<= 7 years	235 (59.49%)
				>7 years	160 (40.51%)
			HIV transmission (Multiple choice)	Sharing needles	302 (51.89%)
				Sexual promiscuity	215 (36.94%)
				Other ways	75 (12.89%)
			Medical History	Cardiovascular disease	79 (20%)
				Physiological disease	61 (15.44%)
				Mental disorder	24 (6.07%)

### Research Methods

#### Questionnaire Design and Reliability Test

A structured questionnaire was designed to sort out and form the following three parts on the basis of a large number of research literatures:

##### Physical activity rating scale (PARS-3)

Revised in accordance with the three-question test method compiled by Liang (1994) and others, Likert’s 5-point scale was adopted to measure the participation level of physical activity from 1 to 5 points. That is the exercise intensity (*what do you think of the intensity of physical exercise you participate in.* According to the options “hardly get a fever every time you exercise, slight fever every time you exercise, a little sweat every time you exercise, more sweat every time you exercise, and sweat every time you exercise” are scored 1–5, respectively), exercise time, (such as, *how long have you been participated in physical activity*. According to the options “Less than 20 min, 21–30 min, 31–40 min, 41–50 min, 51 min or more” are scored 1–5, respectively), and exercise frequency (such as, *how often do you participate in physical activity every week*. According to the options “1 time and below/week, 2–3 times/week, 4 times/week, 5 times/week, 6 times and above/week” are scored 1–5, respectively). Physical exercise score = exercise intensity score × (exercise time score −1) × exercise frequency score, and the score range is 0–100 points. The grade of physical exercise is divided into low physical activity amount ≤19 points, medium physical activity amount 20–42 points, and high physical activity amount ≥43 points. The pre-test of the questionnaire showed high retest reliability and correlation coefficient *r* = 0.82.

##### Internal inhibition scale

The Internal Inhibition Scale compiled and revised by Jiang and Lin (2000) was adopted. This scale contains 25 items, such as *I don’t think I can easily behave well*, *I don’t think I can concentrate on anything easily*, etc., and it was quantified with a Likert 5-point measuring scale. According to the options *very agree, relatively agree, neutral or undecided, not very agree, disagree* are scored 1–5, respectively. After the internal inhibition scale is rotated by the direct skew method, there are three common factors with feature roots greater than 1, including a total of 19 items, and another 6 items are rejected because they have too little contribution and the progressive contribution rate of the three dimensions up to 68.47%. Therefore, the total score of internal inhibition was made up of the addition of 19 items, with scores ranging from 19 to 95. The higher the score is, the stronger the internal inhibition. The Cronbach α coefficient for the total score of internal inhibition was 0.96, which was above the acceptable level. The one-dimensionality of the scale was proved by a confirmatory factor analysis (CFA): x^2^/df = 1.51, RMSEA = 0.04, AGFI = 0.98, CFI = 0.97, TLI = 0.99, IFI = 0.96, GFI = 0.99, it shows that the questionnaire has good structural validity.

##### Drug craving scale

The rug Craving Scale compiled by Jiang and Lin (2000) was adopted. This scale contains 25 items, such as *Taking drugs can make people temporarily forget their worries*, *taking drugs will bring me full of exciting feelings*, etc. A Likert 5-point measuring scale was adopted for quantification, and according to the choice *disagree, not very agree, neutral or undecided, relatively agree, very agree* were, respectively associated with 1–5 points. After the drug craving Scale was rotated by the direct skew method, there were three common factors with feature roots greater than 1, including a total of 21 items, and another 4 items were rejected because they had too little contribution and the progressive contribution rate of the three dimensions up to 64.56%. The three-dimensional Cronbach α coefficient results were in turn: Cronbach α coefficient of medication cognition was 0.92 (9 items), the irrational belief was 0.94 (7 items), and the craving degree was 0.91 (5 items). The total score of drug craving was made up of the addition of 21 items, with scores ranging from 21 to 105 points. The higher the score was, the stronger the drug craving. The Cronbach α coefficient of the total score was 0.96, which was above the acceptable level. The one-dimensionality of the scale was proved by a CFA: x^2^/df = 1.44, RMSEA = 0.05, AGFI = 0.98, CFI = 0.99, TLI = 0.92, IFI = 0.98, GFI = 0.99, which shows that the questionnaire has good structural validity.

#### Mathematical Statistics

SSPS 21.0 was adopted to conduct statistical analysis of the data, the Cronbach α coefficient and CFA were used to test the structure validity and fit of the questionnaire, and Harman’s single factor test method was used to test whether the questionnaire data had a common method deviation. The independent sample *t*-test and single-factor analysis were adopted to conduct demographical variance analysis of internal inhibition of drug addicts with AIDS and drug craving, and the effect size in the independent sample *t*-test was calculated by *Cohen’s d*. For *Cohen’s d* = 0.20 is a small effect size, 0.50 is a medium effect size, and 0.80 is a high effect size. Pearson correlation analysis was adopted to investigate the correlation between physical activity amount, internal inhibition, and drug craving. Amos 21.0 was adopted to build a structural equation model and perform path analysis, and the mediation effect test was carried out using the intermediary inspection process by Gerbing and Anderson (1988) and the Bootstrap program. The fit indices considered in the model evaluation process mainly include x^2^/df, RMSEA, TLI, CFI, GFI, NF, and AGFI. Among them, usually for x^2^/df values less than 5 indicate a better fit (generally 1∼5), for RMSEA values close to 0 indicate a better fit, and for TLI, CFI, GFI, NFI, and AGFI values close to 1 indicate a better fit (Hu and Bentler, 1999; Wen et al., 2004b). Among them, the physical activity amount and the years of drug abuse are observation variables, that is, independent variables. Meanwhile, the drug craving is a latent variable, that is, dependent variable, craving degree, irrational belief, and drug cognition are its observation variables. The significance level of all indicators was set at α = 0.05.

## Results

### Common Method Variance Test

Data collected by questionnaire may have the risk of common method variance, thus the research follows the common method variance test method proposed by predecessors (Zhou and Long, 2004). After data collection, A Harman single factor test was adopted to check common method variance. The results show that there are 10 factors with an eigenvalue greater than 1, and the variance explained by the first factor is 31%, less than the critical standard of 40%, which proves that the common method has no significant variance.

### Demographical Variance Analysis of Internal Inhibition and Drug Craving

In this study, according to the research needs, the drug types are divided into three types: new drugs, traditional drugs and mixed drugs. Among them, traditional drugs mainly include opium, heroin, marijuana, etc. New drugs mainly include methamphetamine (MA), K powder (mainly ketamine), etc. Mixed drug refers to the use of two or more addictive drugs in a certain time period, thereby to form the drug dependence. Similarly, according to the sample characteristics, the drug-using years were recoded into 7 years or less and more than 7 years to further explore the influence of drug use type and drug use years on the internal inhibition and drug craving of addicts.

The independent sample *t*-test results (Table 2) showed that there was no significant difference between male and female drug addicts with AIDS in terms of internal inhibition (*T* = 1.04, *Cohen’s d* = 0.11, *P* > 0.05), and no significant difference in drug craving (*T* = −0.91, *Cohen’s d* = 0.09, *P* > 0.05). In terms of drug-using years, though there was a significant difference in the internal inhibition between addicts of 7 years or less and addicts of more than 7 years (*T* = 4.46, *Cohen’s d* = 0.45, *P* < 0.001), the internal inhibition of the former was higher than the latter, and the drug craving of the former was lower than the latter (*T* = −4.05, *Cohen’s d* = 0.41, *P* < 0.001). The results of one-way analysis of variance showed that in terms of drug types, there was a significant difference in the internal inhibition of drug addicts with AIDS who takes different types of drugs (*F* = 4.06, *P* < 0.01). Among them, the internal inhibition of those who take traditional drugs was significantly higher than that of new drugs and mixed drugs, but there was no significant difference between the new drug and mixed drug addicts (*P* > 0.05). And there was no significant difference in the drug craving (*F* = 2.51, *P* > 0.05).

**TABLE 2:** Demographical variance analysis of internal inhibition and drug craving.

**Variable**	**Internal inhibition**		**Drug craving**	
	***M***	***SD***	***M***	***SD***
Men	66.96	17.94	44.98	18.97
Women	64.98	19.02	46.88	21.24
*t*	1.04		−0.91	
*Cohen’s d*	0.11		0.09	
<= 7 years (A)	69.15	17.66	42.75	19.54
>7 years (B)	60.84	18.87	51.04	20.56
*t*	4.46***		−4.05***	
	A > B		A < B	
*Cohen’s d*	0.45		0.41	
New drug (a)	63.16	18.40	48.96	21.34
Traditional drug (b)	69.17	18.18	44.73	18.11
Mixed drug (c)	64.77	18.88	46.80	21.33
*F* Multiple comparison	4.06** b > a; b > c		2.51	

**P < 0.01, ***P < 0.001.

### Correlation Analysis of Physical Activity Amount, Internal Inhibition and Drug Craving

Correlation analysis showed (Table 3) that there was a significant correlation between the amount of physical activity, internal inhibition and drug craving. Among them, the amount of physical activity was positively correlated with the internal inhibition (*r* = 0.19). There was a significant negative correlation between internal inhibition and drug craving (*r* = −0.38), and there was a significant negative correlation between physical activity and drug craving (*r* = −0.21). Meanwhile, there was a significant negative correlation between drug duration and internal inhibition (*r* = −0.29), and a significant positive correlation with drug craving (*r* = 0.26). It should be noted that since this study mainly explores the relationship between physical activity amount, internal inhibition and drug craving, the relationship between drug duration and physical activity amount will not be further explored. Therefore, the hypotheses H1, H2, and H3 of this study have been confirmed, which provides a good basis for the subsequent testing of the mediating effect of internal inhibition between physical activity and drug craving.

**TABLE 3:** Analysis of the correlation among the physical activity amount, internal inhibition and drug craving (*N* = 395).

**Variables**	***M***	***SD***	**1**	**2**	**3**	**4**	**5**	**6**	**7**
1. Years of drug abuse	7.93	7.40	–						
2. Physical activity amount	24.56	14.87	−0.14**	–					
3. Internal inhibition	65.78	18.59	−0.29***	0.19***	–				
4. Drug craving	46.11	20.35	0.26***	−0.21***	−0.38***	–			
5. Craving degree	8.14	4.59	0.25***	−0.16***	−0.34***	0.81***	–		
6. Irrational belief	14.71	7.28	0.25***	−0.23***	−0.34***	0.84***	0.63***	–	
7. Drug cognition	15.86	8.51	0.21***	−0.14**	−0.30***	0.87***	0.58***	0.55***	–

**P < 0.01 and ***P < 0.001.

### Test of Dual Mediating Effect of Internal Inhibition

According to the procedure of the mediation effect test (Wen et al., 2004a; Preacher et al., 2006), the direct effects of physical activity amount and the years of drug abuse on drug craving are first tested, respectively. Then the model fitting situation and the significance of each path coefficient after adding mediation variables are tested. (1) The fitting indexes of the direct effect of physical activity amount on drug craving were: x^2^/df = 1.88, RMSEA = 0.05, TLI = 0.98, CFI = 0.99, GFI = 0.99, NFI = 0.98, the direct effect of physical activity amount on drug craving was significant (β = −0.23, *SE* = 0.01, *P* < 0.001). (2) The fitting indexes of the direct effect of the years of drug abuse on drug craving were: x^2^/df = 0.45, RMSEA = 0.01, TLI = 0.99, CFI = 1.00, GFI = 0.99, NFI = 0.98, the direct effect of the years of drug abuse on drug craving was significant (β = 0.31, *SE* = 0.03, *P* < 0.001).

**FIGURE 2:**
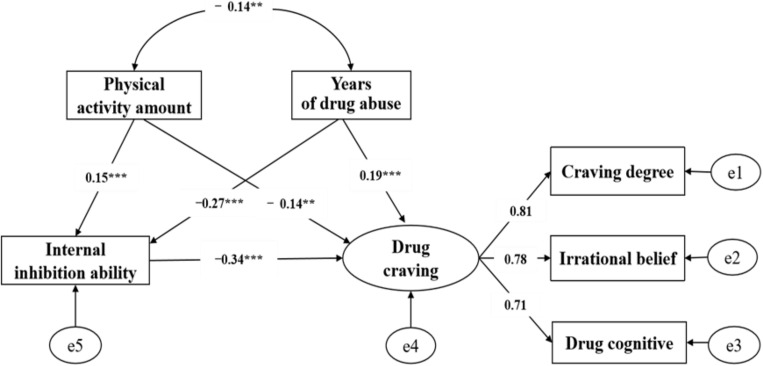
Model diagram of dual mediating effect of internal inhibition.

A mediation variable was added of internal inhibition among physical activity amount, the years of drug abuse, and drug craving, meanwhile a correlation between physical activity amount and the years of drug abuse was established according to Pearson’s correlation analysis results. The fitting indexes of the model in Figure 2 were: x^2^/df = 0.64, RMSEA = 0.01, GFI = 0.99, TLI = 0.99, CFI = 0.99, AGFI = 0.98, NFI = 0.99. The direct effect of physical activity amount on internal inhibition was significant (β = 0.15, *SE* = 0.06, *P* < 0.001), the direct effect of the years of drug abuse on the internal inhibition was significant (β = −0.27, *SE* = 0.12, *P* < 0.001), and the direct effect of internal inhibition on drug craving was significant (β = −0.34, *SE* = 0.01, *P* < 0.001). It was worth noting that after adding the mediation variable, the path coefficient between the physical activity amount and the drug craving decreased from the original (β = −0.23, *SE* = 0.01, *P* < 0.001) to (β = −0.14, *SE* = 0.01, *P* < 0.01); Besides, the path coefficient between the years of drug abuse and the drug craving decreased from the original (β = 0.31, *SE* = 0.03, *P* < 0.001) to (β = 0.19, *SE* = 0.03, *P* < 0.001). But both path coefficients reached a significant level, indicating that the internal inhibition played a partial mediation role between physical activity amount and drug craving, as well as the years of drug abuse and drug craving, that is, the internal inhibition had a double mediation effect. The Bootstrap intermediary test found that the 95% confidence interval of the mediation path “PAV→IIA→DC” was (−0.09, −0.02), and the effect value was −0.05; The 95% confidence interval of “YDA→IIA→DC” was (0.03, 0.11), the effect value was 0.09, and none include 0 (see Table 4 for details). Therefore, the hypotheses H4 and H5 of this study are confirmed.

**TABLE 4:** Statistical table of pathway coefficient (*N* = 395).

**No.**	**Pathway**	**Boot SE**	**CR**	**Direct effect**	**Indirect effect**	**Bootstrap 95%(CI)**
1	PAA→DC	0.01	−2.78	−0.14		(−0.18, −0.12)
2	IIA→DC	0.01	−6.31	−0.34		(−0.37, −0.25)
3	PAA→IIA	0.13	3.21	0.15		(0.11, 0.23)
4	YDA→IIA	0.12	−5.57	−0.27		(−0.28, −0.19)
5	YDA→DC	0.03	3.59	0.19		(0.14, 0.22)
6	PAA→IIA→DC				−0.34 × 0.15 = −0.05	(−0.09, −0.02)
7	YDA→IIA→DC				−0.34 × (−0.27) = 0.09	(0.03, 0.11)

*PAA, Physical activity amount; IIA, Internal inhibition ability; YDA, Years of drug abuse; DC, Drug craving.*

## Discussion

The purpose of this study is to explore the main factors that affect the drug cravings of drug addicts with AIDS and to reveal the internal relationships between these factors. We found: (1) The physical activity amount of drug addicts with AIDS was positively correlated with drug cravings; (2) The internal inhibition was negatively correlated with drug cravings; (3) The physical activity amount was negatively correlated with internal inhibition; (4) The internal inhibition plays a partial mediating role between the physical activity amount and the drug craving. (5) The internal inhibition also plays a partial mediating role between the years of drug abuse and drug cravings of drug addicts with AIDS.

### Difference Analysis of Internal Inhibition and Drug Craving

This study found that there is no gender difference in the internal inhibition and drug craving of drug addicts with AIDS. No matter male or female, once drug addiction occurs, it is difficult to restrain the drug impulse generated by drug stimulation and the strong desire for drugs psychologically. Fox et al. (2006) found in the general study of cocaine addicts that there was no significant gender difference in subjective anxiety and drug craving among cocaine addicts, but further research and discussion were needed among drug addicts with AIDS. At the same time, this study found that there was significant difference for internal inhibition and drug craving of drug addicts with AIDS in different drug types, a traditional drug addict’s internal inhibition is significantly higher than that of new drugs and mixed drug addicts, and the drug craving for the traditional drug is lower than that of the new drug, but for mixed drug addicts there was no significant difference. In fact, compared with traditional drugs, a large number of synthetic new drugs have highly spiritual dependence and hallucinogenic effects, which can produce more strongly, more permanent, irreversible damage the brain of drug addicts, which will directly impair the addict’s cognitive executive functions such as inhibition and control, and thus lead to strong drug use impulse and corresponding drug-seeking behavior (Wang et al., 2016). Moreover, due to their strong central excitation, new drugs can easily stimulate the sexual desire of drug users, which increases the frequency of sexual behavior and the incidence of unsafe sex, and even induce sexual disorder. Thereby to directly or indirectly increase the risk of HIV and sexually transmitted diseases (STD) transmission (Colfax and Guzman, 2006; Passaro et al., 2015), and there is no gender difference in it (Xie et al., 2019). Thus, new drug addicts are more dangerous in this sense, possibly due to their lower internal inhibitions and higher drug craving.

Besides, this study found that drug addicts with AIDS with drug-taking years of more than 7 years were significantly lower in terms of internal inhibition than those with that of 7 years or less. In terms of drug craving, those who take drugs for more than 7 years had significantly higher drug craving than those of 7 years or less, which suggests that there may be a *time node* effect on the internal inhibition and drug craving of drug addicts with AIDS. It may be more difficult to control their behavior after a certain period of drug use. However, at present for drug addicts with AIDS, the time node boundaries of the drug-taking year remain uncertain, although Huang et al. (2019) proposed that there were drug-taking years differences in physical health for drug addicts, the difference nodes for male appear 7 years, the difference node for the female is 12 years, but the time node related to internal inhibition and drug craving needs to be further explored. Found in the study of other STDs, for those who take drugs for more than 5 years, the positive rate of syphilis and hepatitis c virus (HCV) were higher than those who take drugs for 5 years or less, this may be due to increase of age, length of drug use. The possibility of high-risk sexual behavior for drug addicts is increasing (Xu et al., 2017), so the risk of infection, syphilis, and HCV infectious diseases will increase.

### Direct Effect of Physical Activity to Drug Craving

Similar to many previous studies, correlation analysis showed a negative correlation between physical activity and drug craving among drug addicts with AIDS. Linke et al. (2013) found that participation in physical activity can effectively prevent or alleviate the negative emotions of addicts when quitting smoking and reduce their craving for cigarettes. In a study of marijuana and opioid withdrawal populations, drug withdrawal cravings were significantly reduced after aerobic exercise intervention (6 months, 3 times/week, 2 h/time) (Roessler, 2010). Hillman and Drobes (2012) believe that participating in aerobic exercise can, to a certain extent, repair the impaired cognitive control ability of drug abusers, enhance the ability to inhibit the impulse to use drugs, and thus achieve the effect of reducing drug craving.

In addition, Kulig et al. (2003) conducted a study on the relationship between the level of physical activity and drug use, and found that among young men and women, those with higher levels of physical activity and participation in sports teams showed lower rates of drug use. Strickland et al. (2016) pointed out that participation in exercise can enhance the ability of dopaminergic signaling, especially in the *reward* pathway, which can effectively reduce drug overuse. Meanwhile, autonomic exercise can improve the release of dopamine in striatum and the level of dihydroxyphenylacetic acid, a dopamine metabolite in midbrain, while treadmill exercise can increase the extracellular dopamine level. Similarly, Chinese scholars believe that aerobic exercise, as an auxiliary means of rehabilitation, plays an important role in alleviating withdrawal syndrome and reduce drug craving (Wang and Zhou, 2014; Zhao et al., 2018). Wang and Zhu (2017) found through empirical research that regular participation in aerobic exercise can effectively improve the physical fitness of methamphetamine-dependent patients and reduce drug craving. However, limited by the particularity of the sample, there are few similar studies on drug addicts with AIDS at present, and relevant empirical studies are even more rare. However, since there has been no direct report that HIV affects cognitive function and reward pathways, it can be inferred that there should be no significant differences in the internal inhibition or drug craving of general drug addicts and drug addicts with AIDS. Therefore, the study suggests that physical activity can effectively reduce drug craving in drug addicts should be equally applicable to HIV-positive drug addicts.

### Test of Dual Mediating Effect of Internal Inhibition

In this study, the internal relationship among variables was explored by a structural equation model. It was found that the amount of physical activity could positively affect internal inhibition, and internal inhibition could negatively affect the drug craving. This was consistent with previous studies, which show that the amount of physical activity is positively correlated with the self-control of smokers in college students (Zhu et al., 2014). Relevant brain areas responsible for internal inhibition and higher cognitive functions, such as the anterior cingulate, dorsolateral prefrontal cortex and the auxiliary motor area, can be optimized to a certain extent through physical activity, thus to promote the enhancement of inhibition ability for drug addicts (Zhao et al., 2017). It can be concluded that physical exercise can effectively improve the cognitive function of drug addicts, and enhance internal inhibition. In addition, in the field of drug addiction, internal inhibition is closely related to drug craving. Self-control theory indicates that individuals with low self-control are more likely to have corresponding drug craving and abuse behaviors, while individuals with high self-control tend to show less substance abuse behaviors (Tarantino et al., 2015). Similarly, long-term use of meth will lead to abnormal activation of the prefrontal cortex, which will damage the internal inhibition function of users and lead to abnormal cognition and behavior (Wang et al., 2016). Liu (2008) studied drug addicts with AIDS and found that there was a significant negative correlation between their internal inhibition and drug craving, that is, the higher the internal inhibition is, the lower the drug craving they have. However, the study did not examine the important role of physical activity in drug addicts with AIDS. Therefore, this study established a pathway mechanism among physical activity, internal inhibition and drug craving.

In this study, the internal inhibition was used as a mediating variable, and it was found that internal inhibition played a partial mediating role between the physical activity amount and the drug craving. The mediation effect reached a significant level, but it was worth noting that the mediation effect power was only −0.05. This may be due to the long-term use of drugs, which leads to serious damage to the relevant brain regions of the individual’s inhibitory function (Nestor et al., 2011; Kogachi et al., 2017), it was difficult to fully improve the suppression function and effectively reduce the drug craving of addicts through single or short-term physical activity alone. But it should be seen that the application potential of sports detoxification was extreme, previous studies have shown that proper physical activity can not only stimulate brain nerves and improve brain cognitive function, but also effectively reduce the craving for drugs in drug addicts (Xu et al., 2014). After aerobic exercise, the inhibition dysfunction of meth addicts was significantly improved, and the inhibition ability was improved, which in turn significantly reduced the addicts’ drug craving and reduced their relapse tendency (Wang Y. Q. et al., 2015; Wang et al., 2016), and this was consistent with our previous research (Wang et al., 2019). It should be noted that the intensity of physical exercise has always been controversial among the mediating effects of internal inhibition. The results of the pathway model of this study show that physical activity is linearly related to the internal inhibition and drug craving of drug addicts with AIDS, that is, the greater the physical activity amount, the stronger the internal inhibition and the lower the drug craving is. Similar results have been found in college students who are dependent on smoking. Studies have shown that the amount of physical exercise is positively correlated with the self-control of college students who are dependent on smoking, but negatively correlated with their dependence on smoking. In other words, the higher the amount of physical exercise is, the stronger their self-control is, and the weaker their dependence on smoking is (Zhu et al., 2014). In empirical studies, when conducting a Stroop test among meth addicts by using functional near-infrared spectroscopy (fNIRS), Rong et al. (2019) found that compared to medium intensity aerobic exercise, high intensity exercise was more beneficial to increasing the oxyhemoglobin concentration of left dorsolateral prefrontal cortex (left DLPFC) and right ventrolateral prefrontal cortex (right VLPFC) than medium intensity aerobic exercise. And activity of left DLPFC and right VLPFC in the high-intensity group increased significantly before and after aerobic exercise. However, due to the particularity of drug addicts with AIDS, the duration of exercise should be strictly controlled when they have high intensity physical exercise. This is because when an individual is in a state of exhaustion during a long period of high intensity exercise, it will cause a sharp decrease in cerebral blood oxygen value, increase impulse error tendency, and reduce the correction efficiency of incorrect activation (Dietrich and Audiffren, 2011), thus easily leading to the impairment of cognitive function (Chmura and Nazar, 2010). Therefore, formulating a scientific and effective exercise prescription was the key to establishing a long-term mechanism of exercise detoxification.

In other words, our study also found that the internal inhibition also played a partial mediating role between the years of drug abuse and drug craving. Similarly, the mediation effect reached a significant level, but it was worth noting that the mediation effect power was only 0.09. That is, the years of drug abuse can not only directly affect the drug craving of addicts, but also have an indirect effect on drug craving to a certain extent through the intermediary effect of internal inhibition. We speculate that with the increase of drug addiction years, the cognitive inhibition function of the brain of addicts has been damaged by drugs for a long time, which leads to the gradual decline of internal inhibition and further makes it more difficult to suppress the craving for drugs. This may be the important reason why drug addiction is difficult to quit and produces the repeated relapse behavior. Studies have shown that after long-term drug use, addicts will become more dependent on drugs and their physical tolerance to drugs will gradually increase (Jiang, 2006; Chen Y. L. et al., 2017), the degree of drug dependence will also be higher. At the same time, with the extension of drug-taking years, the effects of non-injecting drug use such as oral inhalation gradually fail to meet the strong drug needs of drug users, and 61.84% of non-injecting drug users are likely to convert to injecting drug use within 1–8 years (Zhao et al., 2007), which greatly increases the possibility of AIDS among drug addicts. Thus, drug-taking years may be a potential cause for drug addicts to spread infectious diseases such as AIDS. The above-mentioned results indicate that internal inhibition plays a dual mediating role in the whole pathway model, suggesting that different prescriptions of physical activity should be formulated according to the difference of drug-taking years of drug addicts with AIDS during physical exercise, which may achieve better rehabilitation effects.

## Conclusion and Prospect

### Conclusion

1) There is no gender difference in the internal inhibition and drug craving of drug addicts with AIDS, and there are significant differences in the internal inhibition and drug craving of drug addicts with different drug use types and years.
2) Among drug addicts with AIDS, there was a significant correlation among the years of drug abuse, the physical activity amount, internal inhibition, and drug craving. Among them, the years of drug abuse and the physical activity amount can not only directly affect the drug craving but also indirectly affect drug craving to a certain extent through the mediation effect of internal inhibition.
3) High-intensity physical activity seems to be more effective in improving the internal inhibitions of drug addicts with AIDS and reducing drug craving. Meanwhile, the longer the drug use period, the lower the addict’s internal inhibition, and the higher the drug craving.
4) Through the construction of the mediation effect model, it has revealed to a certain extent that during exercise rehabilitation treatment, attention should be paid to the difference in the years of drug abuse among drug addicts with AIDS, and the formulation of the different load of physical activity prescriptions may be more conducive to reducing the drug cravings of addicts.

### Limits and Prospects

This study has identified the main factors affecting drug craving in drug addicts with AIDS, and further explored the dual mediating effect of internal inhibition between physical activity and drug craving, as well as between drug-taking years and drug craving through structural equation model. However, the study still has the following deficiencies:

1) Due to the adoption of cross-sectional study, direct evidence of causal relationships between variables cannot be obtained, and further studies in cross-lag design and experimental intervention are needed in the future.
2) This study focuses on the dual mediating effect of internal inhibition, and more mediating or regulating variables can be explored in the future.

3) This study does not discuss other disease characteristics of drug addicts with AIDS alone (such as cardiovascular disease, mental disease, etc.), subsequent studies can further explore whether addicts with different disease characteristics have differences in the main variables such as physical activity amount, internal inhibition and drug craving.

## Data Availability Statement

The raw data supporting the conclusions of this article will be made available by the authors, without undue reservation.

## Ethics Statement

The studies involving human participants were reviewed and approved by the Southwest University Hospital Ethics Committee. The patients/participants provided their written informed consent to participate in this study.

## Author Contributions

TZ and KW carried out the protocol and questionnaire survey. XC recruited the individuals with drug addicts. MQ and HJ undertook the statistical analysis and graphical representation of the data. JL revised the draft. All authors designed this study and contributed to the article and approved the submitted version.

## Conflict of Interest

The authors declare that the research was conducted in the absence of any commercial or financial relationships that could be construed as a potential conflict of interest.
